# Thoracic endovascular aortic repair for hemolysis 17 years after insertion of classical elephant trunk: a case report

**DOI:** 10.1186/s13019-023-02415-x

**Published:** 2023-11-10

**Authors:** Atsuyuki Mitsuishi, Nobuyuki Hirose, Unpei Okamoto, Tatsuya Noguchi, Juri Kawaguchi, Yujiro Miura

**Affiliations:** 1 Department of Cardiovascular Surgery, Kochi Medical School Hospital, 185-1, Kohasu, Okohcho, Nankoku-Shi, Kochi Prefecture 783-8505 Japan; 2 Department of Cardiology, Izumino Hospital, 2-10-53 Azono, Kitamachi, Kochi Prefecture 781-0011 Japan; 3 Department of Cardiovascular Surgery, University Hospital, Kyoto Prefectual Hospital of Medicine, 465, Kajiicho, Kamigyo Ward, Kyoto, 602-8566 Japan; 4 Department of Cardiology and Geriatrics, Kochi Medical School Hospital, 185-1, Kohasu, Okohcho, Nankoku-Shi, Kochi Prefecture 783-8505 Japan

**Keywords:** Haemolysis, TEVAR, Classical elephant trunk, Aortic arch replacement, Aortic dissection, Calcification

## Abstract

**Background:**

The classical elephant trunk (ET) technique is a very useful surgical procedure; however, haemolysis in the aorta associated with ET has been previously reported. It normally occurs within several years after the surgery, and it is a rare case of rapidly progressing haemolysis 10 or more years after aortic arch replacement with ET.

**Case presentation:**

A 53-year-old man with a history of Stanford type A aortic dissection (DeBakey type Is), who was treated with total arch aortic replacement and aorto-femoral bypass using a prosthetic graft 17 years ago, developed severe progressive haemolytic anaemia. The ET used for the initial surgery was narrowed, and mechanical haemolysis was suspected. We assumed that progressive mechanical haemolysis occurred because of degeneration of the prosthetic graft. Thoracic endovascular aortic repair was performed, and haemolysis and anaemia were mitigated postoperatively.

**Conclusions:**

Haemolysis occurred 17 years after the initial surgery with ET. When haemolysis is suspected in a patient with ET, it must be identified as a cause of haemolysis even if 10 years or more have passed since the ET was inserted. To prevent this complication, attention should be paid to an appropriate ET length and diameter to avoid folding of the ET, particularly when the true cavity diameter is small.

## Background

The classical elephant trunk (ET) technique is a very useful surgical procedure; however, haemolysis in the aorta associated with ET has been previously reported. It normally occurs within several years after the initial surgery, and it is a rare case of rapidly progressing haemolysis 10 or more years after aortic arch replacement with ET.

## Case presentation

**Fig. 1:**
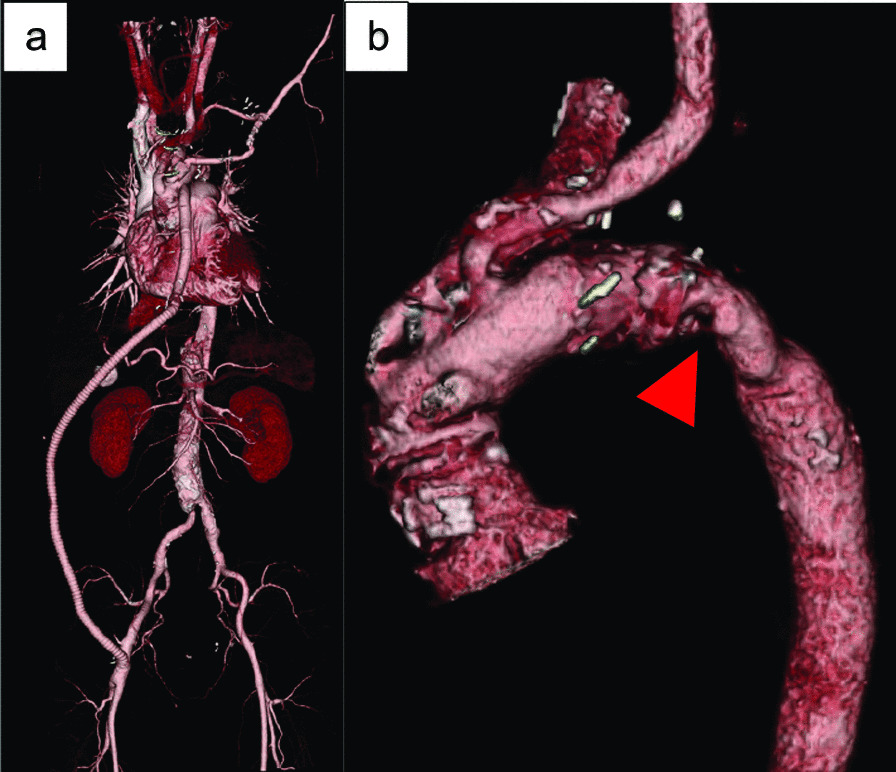
3DCT. **a** Aorto-femoral bypass **b** Stenosis within Elephant Trunk (Red arrows)

A 53-year-old man with chief complaints of chest pain and right lower extremity numbness was diagnosed with Stanford type A aortic dissection (DeBakey type I), with a re-entry tear 3 cm distal to the subclavian artery and decided to undergo emergency surgery at another hospital. The right femoral artery (FA) was not palpable. Through median sternotomy, arterial cannulas were placed in the right axillary artery (AX) and right FA, with an 8-mm branch of a 24-mm prosthetic woven graft (Hemashield Woven Aortic Branch, Angled 4 Branch Graft, GETINGE, Sweden). Venous cannulas were placed into the superior and inferior vena cava. An 8-mm prosthetic vascular conduit was placed in the right AX to facilitate selective cerebral perfusion (SCP). After aortotomy, the primary entry tear was located at the distal to left subclavian artery (LSCA). SCP was initiated through the brachiocephalic artery (BCA), left common carotid artery (LCCA), and right AX conduit. The descending aorta at the tear was narrow, and an 18-mm woven graft (Intergard Woven, Straight, GETINGE, Sweden) was selected to make a 5 cm ET. Distal anastomosis was performed near the left subclavian artery using an outside felt strip. Blood supply to the lower body was started from the side branch. In case of proximal anastomoses, an outside felt strip and inside 8-mm strip of autologous pericardium were used. After removing the cross clamp, the cervical branches were anastomosed. Cardiopulmonary bypass was withdrawn, and decannulation was performed. However, the left and right FA pressures were approximately 50 mmHg lower than that of the aortic root. The pressure of the right AX did not decrease, suggesting ET anastomotic stenosis. Subsequently, transoesophageal echocardiography showed stenosis at the graft anastomosis in the descending aorta, which was dilated with an occlusion balloon; however, lower extremity blood pressure did not improve. Aorto-femoral bypass was performed through subcutaneous tissue from the 10-mm side branch of the 24-mm aortic graft to the right FA with a ring graft (GORE® INTERING® Vascular Graft, GORE, USA; Fig. 1a). Following bypass, the right FA was palpable and left FA pressure increased, even though stenosis at the distal graft anastomosis persisted. The postoperative course was uneventful. Although postoperative magnetic resonance imaging showed a stenotic lesion of approximately 4 cm in size within the graft from the distal anastomosis, the patient was discharged as there were no haemolytic findings.

**Fig. 2:**
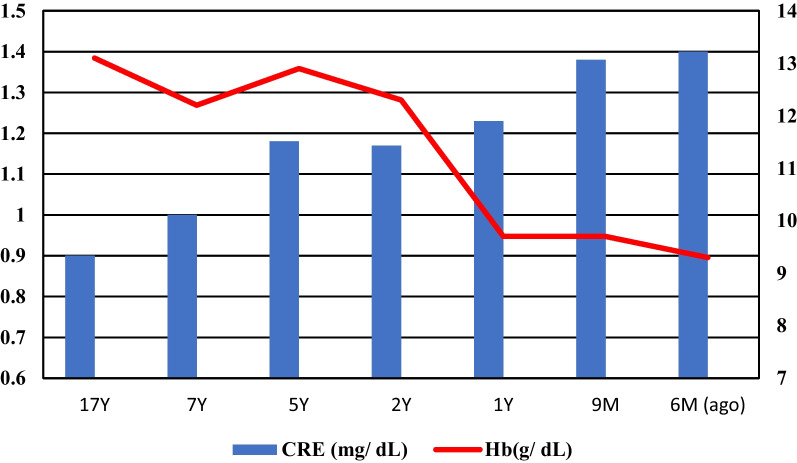
Time course of creatinine (Cre) and Hemoglobin (Hb). *Y* Year, *M* Month

His haemoglobin was approximately 12 g/dL at 16 years after the initial surgery, though it rapidly decreased to 8 g/dL in approximately 1 year even though the cardiologists prescribed beta blockers. His renal function continually deteriorated (Fig. 2), and exertional respiratory distress was observed. Similar to the initial surgery, CT revealed stenosis (Fig. 1b) as the ET and AX bypass were patent. Based on the patient’s blood analyses (Table 1), chronic intravascular haemolysis caused by mechanical damage owing to the stenotic ET was suspected. Moreover, renal dysfunction was considered to be the effect of long-term haemolysis. If not treated may lead to progressive renal dysfunction. We decided to perform diagnostic treatment with TEVAR. The left FA was used, and the prosthetic graft of the aorto-femoral bypass was exposed from the right abdomen. The guidewires were inserted in these two locations. Aorto-femoral bypass to the lower limbs was blocked, and the pressure gradient across the stenosis was 60 mmHg. Transoesophageal echocardiography revealed flow in the false lumen; however, compression of the graft by a bulging false lumen was not observed. A balloon catheter (Medtronic REL46 Reliant Stent Graft Balloon Catheter 12Fr, Medtronic, United State) was inflated, which slightly widened the stenosis, and we performed the ballooning aggressively and repeatedly till no more dilation was possible, avoiding rupture of the suture line due to expanding stenosis.

**Table 1:** Blood test on admission

T-bil	1.1 mg/dL	Hb	8.0 g/dL
D-bil	0.4 mg/dL	Ht	23.6%
ALT	12 U/L	Ret	7.3%
LDH	1336 U/L	Schistocyte	( +)
DCT	(–)	EPO	33.3 mIU/ml
ICT	(–)	Hp	< 10 mg/dL

*T-bil* total bilirubin, *D-bil* direct bilirubin, *ALT* alanine aminotransferase, *LDH* lactate dehydrogenase, *DCT* direct Coombs' test, *ICT* indirect Coombs' test, *Hb* hemoglobin, *Ht* hematocrit, *Ret* Reticulocyte, *EPO* erythropoietin, *Hp* haptoglobin

**Fig. 3:**
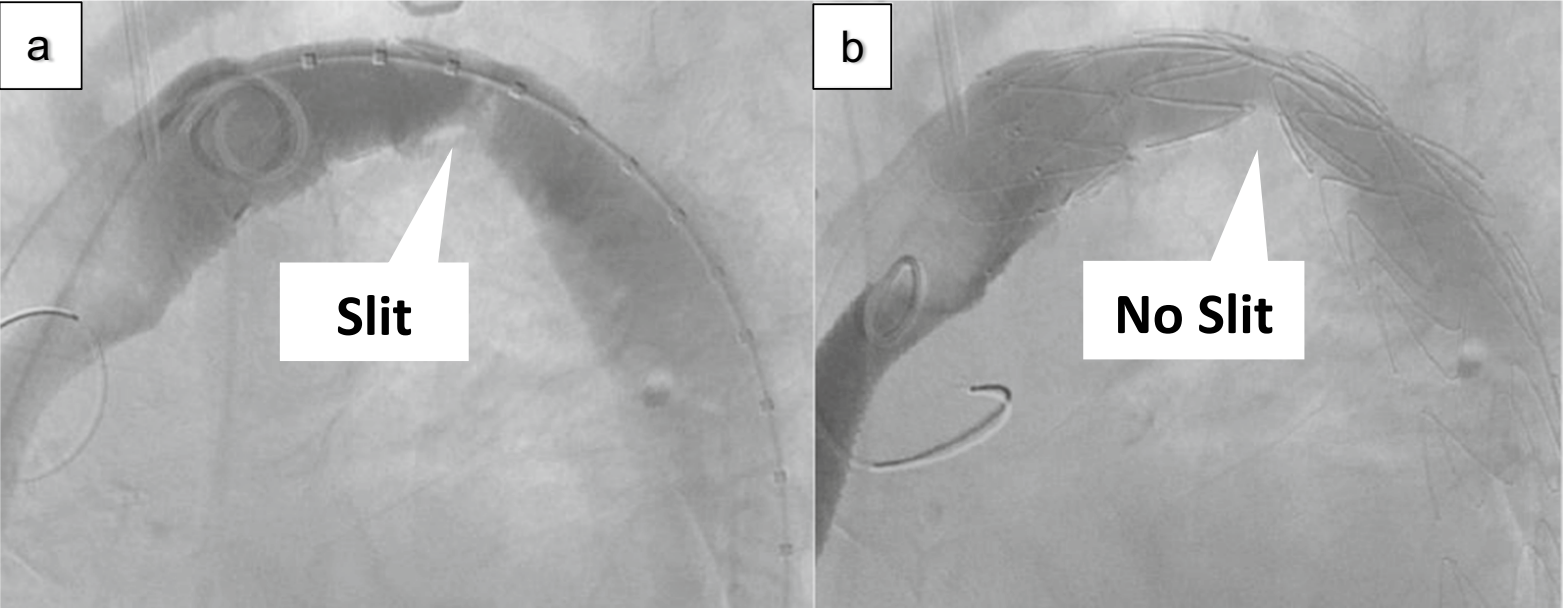
Slit of bending graft **a** Before TEVAR, **b** After TEVAR

**Fig. 4:**
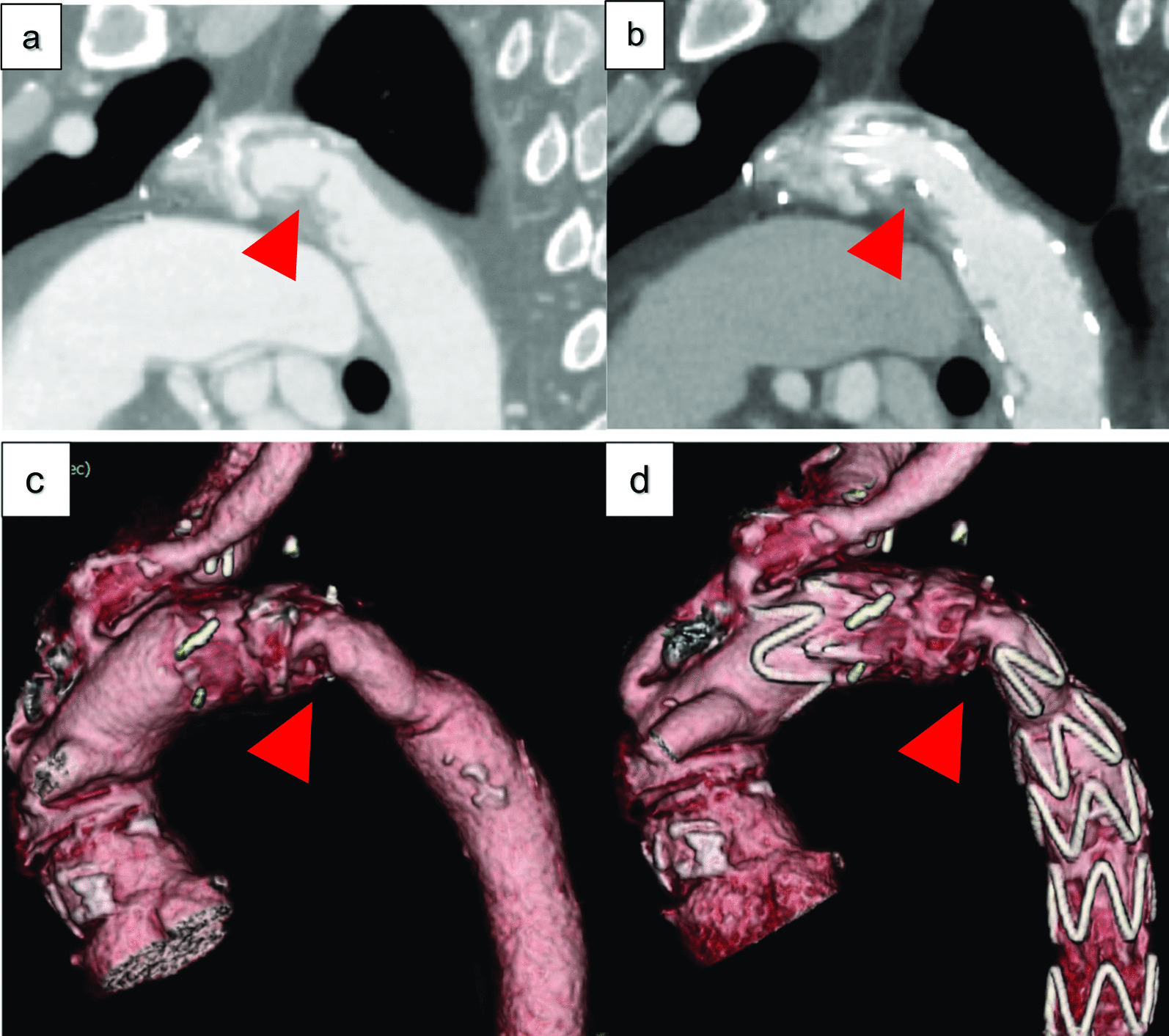
Preoperative and postoperative computed tomography. Narrowing artery within Elephant Trunk (Red arrows). **a**, **c** Before TEVAR, **b**, **d** After TEVAR

Thereafter, the main endovascular graft body ZTA 28–28-155 (COOK Zenith Alpha®, COOK® MEDICAL, USA) was inserted from the left FA and deployed immediately distal to the orifice of LCCA (Zone 3 TEVAR). After the stenosis was carefully enlarged, balloon dilatation was performed at the stenosis (Fig. 3a, b). Subsequently, the pressure gradient improved to 50 mmHg. The operative time was 100 min, and the estimated blood loss was 50 mL. Three days postoperatively, dyspnoea on exertion improved, and five days postoperatively, Hb levels increased. On postoperative axial CT, the largest area of the minor axis diameter of the stenotic ET was dilated from 5 to 11 mm (Fig. 4a, b). The patient was discharged seven days postoperatively. There was no recurrence of anaemia two years postoperatively. This study was approved by the ethics review board of our hospital and conforms to the declaration of Helsinki. The patient provided informed consent for publication of this study.

## Discussion

Symptomatic intravascular haemolysis after prosthetic aortic graft replacement with the ET is rare (Table 2) [1]. Ayaki et al. reported two cases of haemolytic anaemia caused by the bending and folding of ET within a year postoperatively; in both cases, haemolysis was resolved by reoperation [1]. One study reported a case of haemolytic anaemia occurring five years after total aortic arch replacement with an ET due to type A aortic dissection [2]. Haemolysis was caused by graft stenosis of the ET, and TEVAR was performed in the narrowed ET cavity to improve haemolysis. The case presented herein is similar; however, unusually, haemolysis occurred 17 years postoperatively. Infection, pseudoaneurysm, false luminal dilatation, and external pressure on ET, which could be the underlying causes of stenosis and haemolytic anaemia, were excluded. Furthermore, the diameter at stenotic site has not changed in 17 years. The reason behind the occurrence of progressive haemolysis 17 years postoperatively is unclear. Although the definite underlying cause cannot be determined, there are two possible explanations: calcification and surface irregularities. Intraoperatively, it was difficult to dilate the balloon, suggesting graft calcification. Mehta et al. reported many cases of prosthetic graft calcification [3]. A prosthetic graft used for 16 years caused calcification and stenosis [4]. Furthermore, vascular graft failures due to graft calcification have been reported [5]. The calcification of prosthetic grafts is caused by adsorption of calcium and phosphate by the polymer surface, with subsequent subsurface crystallisation [6].

**Table 2:** Summary of cases with ET stenosis

Case	Dender	Age	Graft of ET	Length of ET	Management	Time to hemolysis	Time to operation	Reffernce number
1	Male	64	18-mm Polyester	5 cm	Graft replacement	4 months	1 year	1
2	Male	61	20-mm Polyester	2 cm	Stent graft	Within 1 day	In the same admission	1
3	Male	50	24-mm Polyester	N/A	Stent graft	5 years	5 years	2
Our case	Male	53	18-mm Polyester	5 cm	Stent graft	16 years	17 years	

*ET* Classical elephant trunk, *N/A* Not available

The reason haemolysis was improved, even though the pressure gradient was not significantly improved, could be because the surface irregularities in the ET were smoothed out by the stent graft. Accordingly, haemolysis caused by the ET was probably not only because of stenosis but also calcification formed after long-term exposure to turbulence caused by the stenosis. This scenario leads to a vicious cycle, with calcification creating surface irregularities that consequently cause turbulence and further calcification. TEVAR could stop this cycle by smoothening the surface irregularities and reducing turbulence.

ET is a very useful surgical procedure. Excision of the initial flap is useful for creating a larger true lumen for aortic dissection, [1, 7] but attention should be paid to an appropriate ET length [8, 9] and diameter [2] to prevent folding of the ET, particularly when the true cavity diameter is small. This study has some limitations. First, the cause of sudden haemolysis cannot be clearly understood. Second, the distal anastomotic stenosis was not completely cured, and the patient has been followed-up only for two years without haemolysis. The possibility that even a stent graft could constrict again the flow given the residual slit of the aorta should be contemplated. Furthermore, considering that it has taken 17 years after initial surgery for haemolysis to occur and we have serial CTs from 2 years ago because he was lived in different city and followed previous hospital and we couldn’t measure the sections of the graft over time, there is a possibility for recurrence of haemolysis and its symptoms. Third, a 1 mm slice CT cannot indicate clear calcification, so the cause of hemolysis is not the calcification of the artificial blood vessel, but due to kinking of the ET rather than the ET itself. Fourth, the 18 mm prosthesic graft could be considered from the beginning completely insufficient to ensure distal flow to this patient and could provoke earlier blood cells trauma causing hemolysis. Hemolysis may have improved simply by widening the blood vessels with TEVAR. To our knowledge, it is the longest-term report of haemolysis due to ET. Since the number of cases of haemolysis due to ET is small, further research in the future is warranted.

## Conclusion

We encountered a rare case of progressive haemolysis occurring 17 years postoperatively, which was safely resolved via minimally invasive surgery with TEVAR. When haemolysis is suspected in a patient with ET, it must be identified as a cause of heamolysis even if 10 years or more have passed since the ET was inserted. To prevent this complication, attention should be paid to an appropriate ET length and diameter to prevent folding of the ET, particularly when the true cavity diameter is small.

## Data Availability

All data generated or analysed during this study are included in this published article.

## Abbreviations

ET: Classical elephant trunk
TEVAR: Thoracic endovascular aortic repair
FA: Femoral artery
AX: Axillary artery
SCP: Selective cerebral perfusion
LSCA: Left subclavian artery
BCA: Brachiocephalic artery
LCCA: Left common carotid artery
CT: Computed tomography
